# Analysis of the Hip Status of Golden Retrievers in Brazil—A Study of Health and Genetic Improvement

**DOI:** 10.3390/vetsci12080746

**Published:** 2025-08-11

**Authors:** Luiza Pinto Coelho Ribeiro Jardim, Fabiana Michelsen de Andrade, Darilene Ursula Tyska, Jaime Araújo Cobuci

**Affiliations:** Animal Sciences Department, Universidade Federal do Rio Grande do Sul, Av. Bento Gonçalves, 7712 Agronomia, Porto Alegre 91540-000, RS, Brazil; fabiana.michelsen@hotmail.com (F.M.d.A.); darilenetyska@gmail.com (D.U.T.); jaime.cobuci@ufrgs.br (J.A.C.)

**Keywords:** estimated breeding values, canine genetics, genetic trending, dog welfare, hip dysplasia, inbreeding

## Abstract

Hip dysplasia (HD) is an orthopaedic condition of the hip joints with a complex mode of inheritance that has proven difficult to address through traditional breeding practices in dogs, particularly the most common method, which is the selection by individual phenotype. Employing estimated breeding values (EBVs) into the selection would be a more effective method to reduce the prevalence of HD and would also enable the genetic trends to be monitored. The Golden Retriever is a popular large breed in Brazil, with a reported HD prevalence of up to 19.6%. This study aimed to estimate the breeding values (EBVs) of a sample of Golden Retrievers from Brazilian kennels using Bayesian analysis on a pedigree sample of 1686 dogs, 951 of them with known phenotypes. The posterior means of heritability estimated for hip score through the lowest deviance information criterion value model was 0.15 (posterior standard deviation of 0.08). The EBVs ranged from −0.299 to 0.368, with the average accuracy of 39% with values up to 67%. As expected by simple phenotypic selection, the genetic trend concerning hip scores has been stable since 1975. The study provides breeders with a valuable tool to make informed decisions about selecting sires and dams and contribute to long-term genetic improvement in reducing the prevalence of HD in Golden Retrievers.

## Simple Summary

Did you know that many large-breed dogs suffer from hip problems? Hip dysplasia is a condition of the hip joints which can cause pain and lameness. It is influenced by several factors, including the dog’s genetics. Even when seemingly healthier dogs are selected for breeding, little progress has been made in recent years to reduce the problem. With this in mind, this study analyzed one of the most popular breeds in Brazil, the Golden Retriever, to better understand how genetics can help prevent this condition. From data on lineage and X-ray exams of 951 dogs, we estimated individual breeding values, their genetic potential to either reduce or increase this condition in their offspring. We also found that 15% of the variation in hip scores is genetic, and this prediction can be used to guide safer and more effective breeding decisions, complementing other tools already in use. With these data, breeders will be able to make more informed choices and help ensure the birth of healthier dogs in the future.

## 1. Introduction

Canine hip dysplasia (HD) is one of the most common orthopaedic diseases in dogs, characterized by a malformation of the hip that results in laxity of the coxofemoral articulation. It has been an issue for breeders, especially of large breeds [1]. The method used to reduce HD is the selection by sire and dam phenotype, which in this case consists of their hip scores on the Fédération Cynologique Internationale (FCI) scale. However, given the mode of inheritance of HD is multifactorial [2], environmental factors also contribute to variations in the phenotype. For example, a study conducted in the USA comprising 60 breeds has shown a high heritability of 65% for Golden Retrievers [3]. Another study estimated the heritabilities of 28% in France and Sweden and 41% in the UK [4]. Some studies showed lower heritability, such as a Norwegian sample, with a heritability estimate of 17% [5] and a Dutch sample with 18% [6]. A recently published article evaluated different breeds in Norway and detected heritability values ranging from 25% to 58%. In Brazil, only the German Shepherd [7] and the Bernese Mountain Dog [8] were investigated in this matter, with heritabilities for HD calculated at 19% and 30%, respectively. A survival analysis from a cohort using Boxers has shown that, at the age of 8 years, the predicted proportion of Boxers that were free from clinical signs of HD from sires with the lowest mean EBV (estimated at −0.32 in the study) was decreased by only 6%, compared to 13% of the Boxers fathered by sires with the highest mean EBV (estimated at 0.42) [9]. As such, HD is eligible for genetic improvement using estimated breeding values (EBVs), a more accurate estimate of the genetic liability of a trait than the individual phenotype [10], a method already in use in several developed countries [11]. In this context, the study’s objectives are to estimate genetic parameters and breeding values for HD in the Brazilian Golden Retriever population and, as a result, to provide a more accurate method of selection by breeders, helping to reduce the prevalence of the disease in the breed.

## 2. Materials and Methods

The sample started from the pedigrees of 326 Golden Retrievers in reproduction in the years of 2020–2022 provided by ten Brazilian kennels. From each animal, we traced four generations of ancestors and compiled a database comprising 1686 individuals, with an average number of discrete generation equivalents of 3.90, with a maximum of 10.26. The database included 1044 females and 642 males, with birth dates dating back from 1975 to 2021. A total of 951 of those had available phenotypic data. The data used in this study came from pedigrees and hip reports provided by breeders and an open database of the breed [12]. Data of the studied sample are shown in Table 1.

The database contained each individual’s name, pedigree information of four generations, kennel, and year of birth. Other available data included: sex, country of origin, and source of the hip score as the fixed effects used in the analyses, coefficient of inbreeding (F) as covariate and the hip score itself. These scores range from “A” for healthy hips to “E” for severe dysplasia. Dogs with moderate to severe dysplasia are scored as “D” and “E”, respectively. Those with mild dysplasia are scored “C”, while those with close-to-healthy hips are scored “B”. Dogs scored “D” and “E” are not allowed to breed, while those with “C” can only breed with “A” hips and those with “B” can only breed with other “B” hips or healthier “A” hips [13]. As the standard practice, all the X-rays were taken between 12 and 24 months old, and the hip scores were codified as 1 for healthy hips (A score), 2 for fair hips (B score), and 3 for mild dysplasia (C score), as no scores worse than ”C” were present in the sample. The source of the scores (whether were they presented as a signed report from a veterinarian (n = 43) or from the Orthopedic Foundations for Animals (OFA) [14], or if they came from indirect sources such as sites or breeders), was also employed as fixed effect in the estimation of breeding values.

The THRGIBBS1F90 software [15] was used to estimate the variance components and breeding values, with 1,600,000 iterations of the GIBBS Sampler ran with a burn-in period of 900,000 iterations and sampling interval of 100 iterations, following the single-trait model:

(1) y=Xb+Za+e

where *y* is the hip score for the individual, *b* the vector of effects (sex, n = 2; continent of origin, n = 4; source of scores, n = 45) and coefficient of inbreeding, according to the evaluated model, *a* the vector of random additive genetic effects, *e* the vector for random residual effect, and *X* and *Z* the incidence matrices relating the phenotype to *b* and *a*, respectively. The effects of F and $F^{2}$ were included in the model as covariates. The study evaluated twelve different models (Table 2). We used a flat (improper uniform) prior on the standard deviation parameter, defined as p(σ)∝1forσ>0.

Using the final samples generated by the Gibbs sampler, we employed the POSTGIBBSF90 software [15] to derive the posterior means and standard deviations of the EBVs, as well as the posterior distributions of the variance components.

The F values were obtained through CFC software (Coancestry Inbreeding Contribution, Mumbai, India, https://cfcsoftware.com/) considering the total sample of 1686 dogs, calculated using Wright’s individual pedigree-based coefficient of inbreeding [16]. To analyze the effects of the overuse of sires and the impact on the sample’s EBVs, we selected the males who sired the most pups using the Poprep software [17] and who are further addressed as popular sires.

To diagnose convergence and calculate descriptive statistics of variance components, we utilized the BOA package of the R software [18]. The test of credible interval (CI) half-width was employed to monitor convergence, with a value below 0.01 considered acceptable.

For measuring accuracy, we utilized the following formula:

(2) $$\sqrt{1-\frac{SE^{2}}{\left(1+F\right)\cdot \sigma_{a}^{2}}}$$

where SE represents standard error, F denotes the individual inbreeding coefficient, and $\sigma_{a}$ is the estimated additive genetic variance in the population [10].

Statistical analyses were conducted to examine the relationship between the obtained values and each effect. The relationship between F and EBVs, as well as between phenotypes and EBVs, was examined using Spearman correlation.

Additionally, a linear regression analysis was performed to assess the relationship between EBV and year of birth of dogs (genetic trend).

The Kruskal–Wallis test was used to assess differences between sexes, kennels, and origins, with post hoc tests when necessary.

## 3. Results

### 3.1. Population Structure and Inbreeding

The total pedigree database had 1154 inbred dogs (54.49%). The average coefficient of inbreeding (F) for the total sample was 0.04, and 0.07 between the inbreds, with the maximum F value up to 0.33. To account for the effects of sex, origin, source of hip scores, and inbreeding in the estimation of genetic values, a total of twelve different models were evaluated (Table 2 and Table 3). When compared to their counterparts, the models with the added effects of F and (F)^2^ (quadratic regression) had a worse DIC than those that considered only F (linear regression). However, a better adjustment was obtained when the effect of inbreeding (linear or quadratic) was disregarded (Table 3). The influence of inbreeding (F) on the phenotype (HD) and EBV was demonstrated to be statistically non-significant on both correlation tests and regression analysis.

### 3.2. Estimated Breeding Values

Models that took the source of scores into consideration outperformed their parallels that did not incorporate this effect, showing a higher adjustment with their lower deviance information criterion (DIC) values. The best fitting model was Model 1 (DIC = −4059.8707) and had “source of score” as its only fixed effect. The elected model showed a low heritability of 15% (0–30%) and a genetic variance of 0.111 (0.001–0.226) (Table 3). The estimated breeding values estimated through the chosen Model 1 had an average of −0.015, ranging from the best value of −0.298 to the worst value of 0.369, identifying the best choices of sires and dams to reduce the trait in the breed as those individuals with lowest values. Furthermore, dogs holding positive breeding values were identified as contributors to worst hip scores in the offspring. The individuals with EBVs had an average accuracy of 39% ranging up to 67%. No significant difference between breeding values of females and males was shown.

**Figure 1:**
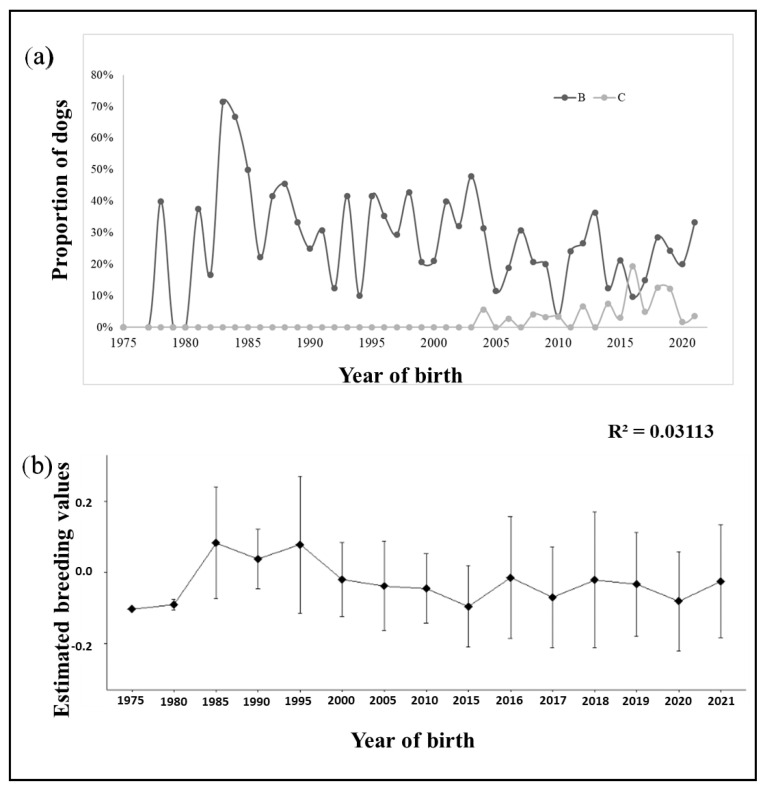
Phenotypic (**a**) and genetic (**b**) trends through the years. Lines “B” and “C” stand for registries of dogs with hips scored “B” (fair hips) and “C” (mild dysplasia), respectively.

Figure 1 depicts both genetic and phenotypic trends for “B” and “C” phenotypes, from 1975 to the 2020s. Figure 1a shows some significant peaks of dogs scored “B” (fair hips) in the 1980, as well as a surge of dogs scored “C” (mild dysplasia) in 2015. As a possible consequence, there was also a slight peak in the breeding values for those periods, as shown in Figure 1b. However, despite these fluctuations, the genetic trend displays a relatively stable pattern over the entire period (Figure 1b; b = −0.0020; R^2^ = 0.03113).

### 3.3. Correlation Between Hip Scores and EBV

**Figure 2:**
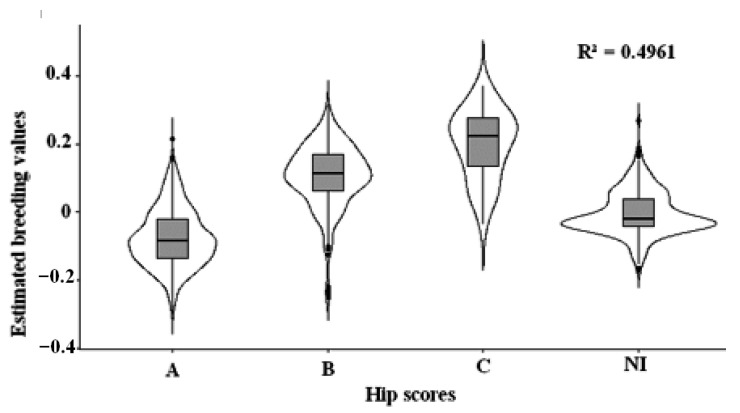
Distribution of estimated breeding values (EBVs) across hip score phenotypes. NI stands for not informed. Horizontal bars demonstrate the median breeding values, while the superior and inferior pieces of the rectangle indicate the inter-quartile range. The areas of the violins represent the density of data in each value of Y-axis, and the dots outside of it are the outliers.

As expected due to low heritability, the relationship between the sample’s phenotypes (hip scores) and breeding values suggests an incomplete correspondence. This is demonstrated in Figure 2 where, while a few animals scored “C” had low EBVs, animals with high, undesirable EBVs were not exclusive to this group, but found in all groups including the supposedly superior “A”. Animals without known phenotype were still able to have their breeding values estimated due to information from their relatives. This kind of demonstration, rarely used in the scientific literature, is of great interest for genetic counseling for breeders, once it makes clear how the phenotype can be a poor tool for genetic improvement of the disease.

The linear regression analysis showed a significant relationship (*p* < 0.01) between the HD scores and the EBVs, with an R-squared of 0.49, explaining 49% of the variance of the latter. However, the remaining 50.39% of the EBV variance is not explained by the phenotype, meaning other factors could influence the EBVs. These data show the importance of preferring breeding values to phenotype in the breeding choices.

### 3.4. Popular Sires

Among the sires, seven were identified as the most influential based on the number of pups sired, as theirs (17 to 52) surpassed those of the other males who were more representative of the average (Table 4). Among them, three individuals (S.5 to S.7) were classified as the least favorable sires based on breeding values, while the other four (S.1 to S.4) were the most favorable ones. Notably, S.5, despite having higher (worse) breeding values, was found to be the second most popular sire in the sample with 29 descendants. Alternatively, among the best sires, S.1 and S.2 had the lowest EBVs in the entire sample. Of the seven popular sires identified, S.2 and S.3 showed the greatest impact on lowering the breeding values in their offspring. It is interesting to see that from these seven popular sires, three of them had their HD scores not available, showing that even the simple control via phenotype is not always correctly used in national dog breeding.

**Figure 3:**
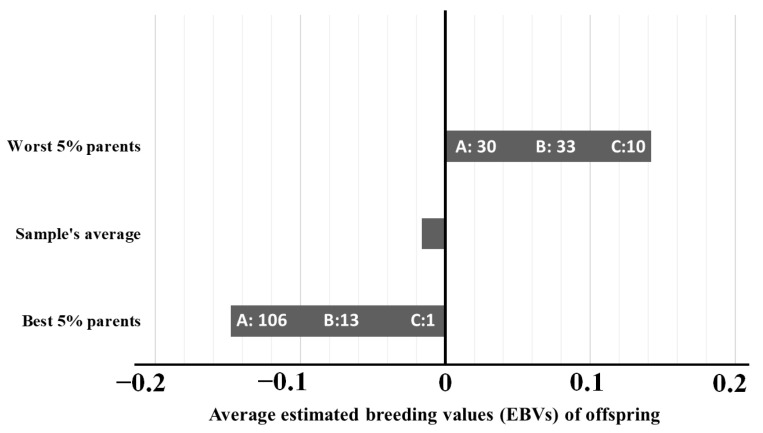
Comparison between the average estimated breeding values (EBVs) of offspring (X-axis) born from the 5% parents with the best (lowest) EBVs, born from the 5% parents with the worst (highest) EBVs, and the average breeding value of the sample. Inside the bars is the total of known hip scores (A, B, and C) from the offspring of each group.

To demonstrate the influence that parents’ EBVs have over the pups’ phenotype and genetic values, two sets of parents were examined apart from the total sample: one comprising the top 5% of parents with best (lowest) breeding values and another with the bottom 5%, with the worst (higher) values. As depicted in Figure 3, the top 5% group had its majority of descendants with hip score “A”, whereas bottom 5% showed a notable amount of “B” and “C” scores within their lineage. The expected influence on offspring genetic values was confirmed for both groups.

## 4. Discussion

Brazil has the world’s second largest dog population, with 55.9 million dogs, trailing only the United States [19]. Although there have been some efforts to discourage dog breeding and promote adoption through the popular slogan “Não compre, adote” (“Don’t shop, adopt”), the number of registries in the country has continued to rise in recent years [20]. Despite the significant population of dogs and the growing industry surrounding them, the scientific understanding of canine genetics and its applications to promote their welfare is limited and underdeveloped in the country. Although some breeders understand the importance of controlling the prevalence of HD for the welfare of purebred dogs, the traditional breeding practice in the country does not consider the genetic information from the extended family of breeding dogs. As a result, the focus remains exclusively on the phenotype of the sires and dams. This selection strategy has not resulted in significant genetic progress, and studies regarding genetic values of hip scores associated with genetic diversity of the Brazilian populations are imperative.

The average inbreeding level of the total sample of Brazilian Golden Retrievers was 0.04, with a maximum of 0.33. In comparison with Leighton’s study on USA-born Golden Retrievers [21], our study had a slightly lower average inbreeding coefficient (0.05 vs. 0.04), but a higher maximum value (0.27 vs. 0.33). Zhang’s study with various breeds showed similar results, with an average F of 0.08 for the Golden Retriever and a maximum of 0.25. The average F and among the inbred dogs in all breeds studied was 0.06, with and a maximum value of 0.37 [22]. While the Brazilian sample’s F was shown to be on par with that of other countries, it does not negate the fact that the maximum values were high, and with 14% of its sample with F over 0.10, it is reasonable to assert it as an issue that should be minimized.

The posterior means of heritability (h^2^) estimates for hip scores was 0.15 (with an additive genetic variance of 0.110 and environmental variance of 0.592), which is consistent with the findings of some previous studies such as Lingas and Klemetsdal (1990) [5] and Lavrijsen (2014) [6], reporting heritabilities of 0.17 and 0.18, respectively. However, other studies, such as Lewis (2013) [10] and Oberbauer (2017) [3], reported higher heritabilities of 0.40 and 0.65 for Golden Retrievers, respectively. In Lewis’s work [10], the additive genetic variance was 0.313 and the environmental variance was 0.126. These differences can be explained by higher genetic variability in the population studied, environmental factors and inclusion of effects such as the coefficient of inbreeding in the elected model (as in Lewis’s study), and the methods used to measure hip scores, such as in Lavrijsen’s work [6], where a closed trio of radiology and/or orthopaedic surgery experts evaluated anonymous radiographs. We suspected that the quality of the source of scores could play a role in the breeding value estimation. Unlike other studies in which x-rays were performed and evaluated by the team [23] or had reliable sources, such as OFA’s database [3] and Brazilian Society of German Shepherd Breeders [7], ours faced challenges with the origin of the information. Therefore, we included the source of score as an additional effect to minimize this influence.

The stability of genetic and phenotypic trends, despite the efforts from breeders, was unfortunately anticipated due to relying exclusively on phenotype in their breeding practices. Several studies, both nationally (rare) and globally, have produced similar results on this subject, with unanimous conclusions that the implementation of EBVs in their breeding programs would effectively hasten the genetic progress [3,24,25,26].

In this study, no significant correlation was found between the F and hip scores or EBVs, which is consistent with Comhaire’s study [27] with Belgium dogs of various breeds. A correlation was reported, however, in Leighton’s study [21] involving German Shepherd Dogs, Labrador Retrievers, Golden Retrievers, and their crosses, with significant correlation for all three breeds. For each 1% increase in their F, a decrease of 0.001 units in their distraction index (DI) was observed, which is the distance between the centre of the femoral head in compression and the centre in distraction by the radius of the femoral head [21]. We believe that the knowledge on breeding values that we gathered from the literature and our own analyses is of huge importance for the field of dog breeding. However, it must also be translated to the end users. Some of our present data may seem obvious to readers with a scientific background, but they provide important points of view that should be used to reach veterinarians and breeders.

Overall, we can observe a reasonable correspondence between phenotype and mean breeding values, given the R-squared value of 0.49 (Figure 2). However, inside each violin plot, there is a broad individual diversity, with EBVs ranging from positive to negative: some animals scored “A” with high EBVs while a few scored “C” with relatively low EBVs. This is consistent with the low heritability, suggesting the phenotype does not represent the EBV accurately. Despite the notable R-squared, there is considerable variability, meaning we cannot rely solely on phenotype, as is often the case with traditional selection method: not only one may select an animal with a good score, but with high breeding values, there is also a risk of overlooking an animal with a less-than-desirable phenotype that could, in fact, contribute to better hip health. We also analyzed the most influential sires selected among the popular sires, based on offspring number, once they have the most significant impact on the population. Some of these popular sires have not had hip scores made available to the public, thereby deviating from the current breeding practice, as is the case of sires S6 and S7: despite their positive breeding values (0.08 and 0.129, respectively), each still sired 25 pups in the sample. This demonstrates the issues associated with the popular sire effect, whereby males are overbred based on their superficial qualities, such as titles and appearance, as is the case with S.6 and S.7, having won several champion titles including in the international category, despite their detrimental impact on canine health and genetic improvement of the disease.

Finally, to illustrate the effectiveness of selection by EBVs, Figure 3 shows phenotypes and average EBVs of the pups from 5% parents with the best values and the 5% parents with the worst values, which makes clear the importance of using the EBV by technicians and breeders.

An important final point which must be approached is the intensity of the selection. While it may seem like a simple solution to remove animals with positive EBVs from the breeding stock, mainly for purebred dogs, it is important to prioritize maintaining genetic diversity in the population. To achieve this, it is necessary to retain as many sires and dams as possible to avoid the risk of an increased coefficient of inbreeding and a lower effective population size. Removing too many animals could worsen existing problems and create unintended consequences, so it is crucial to carefully consider the long-term implications of breeding decisions. Not to mention, caution must be exercised in interpreting the results of this study. It is important to acknowledge that the subjects under investigation are dogs, which are valuable to the breeders who invest in their acquiring, raising, and maintenance. Therefore, any recommendations resulting from this research must be presented with sensitivity and consideration for the potential impact on the breeder and their relationship with their animals. Based on these points, we believe selection intensity should not be as rigid as with production animals, as there is a risk of significant loss of genetic variability, and of some breeders disregarding the research if they feel their priorities are not being respected. Regarding this matter, it would be sensible for breed clubs to provide a selection index based on the union of genetic parameters for HD and F values, as well as other relevant parameters. Another recommendation would be to regulate the number of pups per animal, thereby reducing the popular sire effect and promoting genetic diversity to healthier levels.

Although we are one of the pioneers in a completely unexplored field in Brazil, our analyses have a few limitations. First, there is a small percentage of dysplastic animals in the sample, with only 35 animals scoring “C” and none representing worse scores “D” and “E”. Certainly, our analyses and results would have been far more enriching had we managed to obtain a sample representing the true phenotypic proportions of the breed. However, this is the reality of virtually every other study in the literature, including those with larger samples. Similar to the present study, several authors have highlighted this issue as limitation for their own analyses [3,5,28], presenting this point as an universal limitation, even in countries where genetic improvement in dogs is a well-established field. One of the consequences imposed by this limitation is the wide credible interval (CI) for the genetic parameters, a common occurrence in other works. Nevertheless, since the use of EBVs in mate selection would serve as a substitute for the selection based solely on phenotypes, as much as there may be some uncertainty, it remains more accurate than the traditional method. The third delicate issue involves the eligibility of data collected from public open databases, which was the only alternative to obtain the phenotypic information of part of our sample. It should be noted that, as the only available source for a study of this nature, an effort was made to appropriately adjust the effects based on the source of data (whether it was collected indirectly or directly from the radiology report). The impact of this differentiation was tested in the various models seen in Table 3, and indeed, the best fitting model included source of hip score as its only fixed effect. A considerable portion of these limitations, which, as a whole, can lead to a less-than-desirable accuracy in the estimation of breeding values, could be resolved in the future, if the breeder clubs were to pay more attention to the need for reliable phenotypic reports. Unfortunately, skepticism is frequent among dog enthusiasts, especially in a developing country like Brazil.

## 5. Conclusions

Through Bayesian analysis of multiple statistical models, we identified the most suitable model to predict breeding values for HD for the breeding stock of Brazilian Golden Retrievers. A total of 1252 individual EBVs were obtained, ranging from −0.299 to 0.370, indicating sufficient variability for effective selection. Relying on the proper interpretation of the results, these EBVs can be used by breeders to provide a more accurate tool to guide breeding decisions and to facilitate long-term genetic improvement. In fact, the results of the present study were adapted into an accessible guide for the breeder, with indications of EBVs and explanations in an informal tone on how to proceed with the selection of mates. Given the critical role that dogs play in the lives of millions of Brazilians, it is imperative to invest in scientific research to promote their well-being and improve their living conditions. Enhancing our scientific understanding of dog genetics can help establish more effective policies and regulations to better control the dog breeding industry and ensure the welfare of dogs, from their conception onwards.

## Figures and Tables

**Table 1:** General characteristics of the sample of Golden Retriever in Brazil.

	N
Sample size	
Total phenotyped sample	1686
Sample with phenotype	951
Phenotype–* FCI scores	
Total scores	951
A (=score 1)	667
B (=score 2)	249
C (=score 3)	35
with phenotypes by continent	
South America	238 A|84 B|23 C
North America	256 A|101 B|1 C
Europe	60 A|22 B|2 C
Unknown	113 A|42 B|9 C

* Fédération Cynologique Internationale.

**Table 2:** Fixed effects and covariates used in models to estimate variance components and breeding values of Golden Retrievers in Brazil.

* Model	** Source of Score	Sex	Origin	*** F	(F)^2^
**Model 1**	**X**				
Model 2		X			
Model 3			X		
Model 4	X	X			
Model 5	X			X	X
Model 6	X	X		X	X
Model 7	X		X	X	X
Model 8		X		X	X
Model 9	X	X		X	
Model 10	X		X	X	
Model 11	X			X	
Model 12		X		X	

* The effects were separately evaluated through analysis of variance, each demonstrating *p*-values greater than 0.05. Thus, each combination of these effects was tested. ** This fixed effect is related to the source of the phenotypic data, which was collected in different ways: we attributed a number to each veterinarian we could identify from the radiology reports, computing 43 different professionals, plus direct scores from OFA, and indirect scores from sites such as K9data.com and the dogs’ profile pages on their kennel sites, resulting in 45 levels. *** Inbreeding coefficient.

**Table 3:** Posterior means, posterior standard deviation, 95% Credible interval of heritability components, and DIC for each model.

	Models *			
	**Model 1**	**PSD**	Model 7	PSD
$\sigma_{a}^{2}$	**0.111 (0.001–0.226)**	0.06	0.110 (0.000–0.225)	0.06
$\sigma_{e}^{2}$	**0.592 (0.417–0.774)**	**0.09**	0.617 (0.433–0.812)	0.09
$h^{2}$	**0.15 (0.00–0.30)**	**0.08**	0.15 (0.00–0.29)	0.08
DIC	**−4059.8707**		−3784.9228	
	Model 2	PSD	Model 8	PSD
$\sigma_{a}^{2}$	0.108 (0.000–0.218)	0.06	0.147 (0.039–0.267)	0.05
$\sigma_{e}^{2}$	0.889 (0.322–1.030)	0.09	0.515 (0.369–0.674)	0.08
$h^{2}$	0.15 (0.00–0.32)	0.07	0.22 (0.00–0.39)	0.08
DIC	−3976.0520		−3107.6766	
	Model 3	PSD	Model 9	PSD
$\sigma_{a}^{2}$	0.101 (0.000–0.216)	0.06	0.115 (0.003–0.250)	0.06
$\sigma_{e}^{2}$	0.610 (0.441–0.813)	0.09	0.595 (0.414–0.778)	0.09
$h^{2}$	0.14 (0.01–0.31)	0.08	0.16 (0.00–0.30)	0.08
DIC	−3856.8272		−4030.0710	
	Model 4	PSD	Model 10	PSD
$\sigma_{a}^{2}$	0.144 (0.012–0.257)	0.05	0.108 (0.008–0.229)	0.06
$\sigma_{e}^{2}$	0.507 (0.263–0.659)	0.07	0.602 (0.433–0.798)	0.09
$h^{2}$	0.22 (0.07–0.38)	0.07	0.15 (0.00–0.34)	0.08
DIC	−3197.3772		−3945.1840	
	Model 5	PSD	Model 11	PSD
$\sigma_{a}^{2}$	0.111 (0.003–0.224)	0.06	0.108 (0.001–0.224)	0.06
$\sigma_{e}^{2}$	0.603 (0.433–0.804)	0.09	0.614 (0.436–0.812)	0.09
$h^{2}$	0.15 (0.02–0.33)	0.08	0.15 (0.00–0.29)	0.08
DIC	−3926.4168		−3821.3597	
	Model 6	PSD	Model 12	PSD
$\sigma_{a}^{2}$	0.106 (0.000–0.221)	0.06	0.145 (0.038–0.253)	0.05
$\sigma_{e}^{2}$	0.610 (0.431–0.806)	0.09	0.511 (0.366–0.671)	0.08
$h^{2}$	0.15 (0.00–0.29)	0.08	0.22 (0.06–0.36)	0.07
DIC	−3843.6259		−3155.3374	

$\sigma_{a}^{2}$—additive genetic variance; $\sigma_{e}^{2}$—environmental variance; $h^{2}$—heritability; DIC—deviance information criterion; PSD—posterior standard deviation. * Effects used in each respective model are listed in Table 2.

**Table 4:** Comparison of breeding values between the seven most influential sires.

	Number of Offsprings	** EBV	EBV of Offspring	Accuracy	Hip Score
Best males					
S.1	16	−0.206	−0.03 ± 0.32	0.34	A
S.2	17	−0.135	−0.01 ± 0.31	0.51	* NI
S.3	52	−0.064	−0.02 ± 0.31	0.62	A
S.4	15	−0.022	−0.01 ± 0.29	0.68	A
Mean EBV					
of offspring			−0.02 ± 0.30		
Worst males					
S.5	17	−0.002	0.00 ± 0.29	0.65	B
S.6	25	0.080	0.02 ± 0.31	0.57	* NI
S.7	25	0.128	0.06 ± 0.30	0.49	* NI
Mean EBV					
of offspring			0.02 ± 0.30		

* Not informed, ** Estimated breeding values.

## Data Availability

The datasets used and analyzed during the current study are available from the corresponding author upon reasonable request.

## Abbreviations

The following abbreviations are used in this manuscript:

EBV	Estimated breeding value
HD	Canine hip dysplasia
FCI	Federación Cynologique Internationale
CBKC	Confederação brasileira de cinofilia
OFA	Orthopedic foundation for animals
F	Coefficient of inbreeding
DI	Distraction index
USA	United States of America
NI	Not informed
DIC	Deviance information criterion
CI	Credible interval
SD	Standard deviation
PSD	Posterior standard deviation
